# Element Concentrations and Histopathology of Liver and Kidney in West Greenland Ringed Seals (*Pusa hispida*)

**DOI:** 10.3390/ani14121739

**Published:** 2024-06-08

**Authors:** Emilie U. Andersen-Ranberg, Pall S. Leifsson, Frank F. Rigét, Jens Søndergaard, Steen Andersen, Aage Kristian Olsen Alstrup, Rune Dietz, Christian Sonne

**Affiliations:** 1 Arctic Research Centre (ARC), Department of Ecoscience, Faculty of Technological Sciences, Aarhus University, Frederiksborgvej 399, P.O. Box 358, DK-4000 Roskilde, Denmark; ffr@ecos.au.dk (F.F.R.); js@ecos.au.dk (J.S.); rdi@ecos.au.dk (R.D.); cs@ecos.au.dk (C.S.); 2 Department of Veterinary Clinical Sciences, Faculty of Health and Medical Sciences, University of Copenhagen, Dyrlægevej 16, DK-1870 Frederiksberg, Denmark; 3 Department of Veterinary and Animal Sciences, Faculty of Health and Medical Sciences, University of Copenhagen, Ridebanevej 3, DK-1870 Frederiksberg, Denmark; ple@sund.ku.dk; 4 Hunters Science, Rådmandsgade 55, DK-2200 Copenhagen N, Denmark; stan@dk4.dk; 5 Department of Clinical Medicine, Aarhus University, Palle Juul-Jensens Boulevard 99, DK-8200 Aarhus, Denmark; 6 Department of Nuclear medicine & PET, Aarhus University Hospital, Palle Juul-Jensens Boulevard 99, DK-8200 Aarhus, Denmark

**Keywords:** mercury, cadmium, selenium, ringed seals, Qaanaaq, Qeqertarsuaq, kidney, liver, histopathology, Greenland

## Abstract

**Simple Summary:**

The ringed seal is part of the daily diet for local Inuits in Greenland, and therefore, the contents of trace elements are being monitored bi-annually under the AMAP CORE Programme. In this study Hg, Cd and Se concentrations were measured in ringed seal livers, along with pathological changes in both the liver and the kidneys of the seals. The oldest seals had the highest concentrations of the three trace elements, while the content was the same in both sexes. In more than half of the livers, we found mononuclear cell infiltration (94.7%) and portal cell infiltration (68.4%), while glomerular mesangial deposits (54.1%) were the predominant finding in the kidney.

**Abstract:**

Ringed seals are consumed in Greenland and are therefore included as a key biomonitoring species with the focus on pollution exposure and health effects. Ringed seals in Central West Greenland (Qeqertarsuaq) and in North West Greenland (Qaanaaq) were analyzed for metal concentrations in the liver and histological changes in the liver and kidney. The mean liver concentration of mercury in Qaanaaq was 3.73 ± 5.01 µg/g ww (range: 0.28–23.29 µg/g ww), and the mean cadmium concentration was 7.80 ± 8.95 µg/g ww (range: 0.013–38.79 µg/g ww). For Qeqertarsuaq, the liver concentration of mercury was 1.78 ± 1.70 µg/g ww (range: 0.45–8.00 µg/g ww) and the mean cadmium concentration was 11.58 ± 6.32 µg/g ww (range: 0.11–25.45 µg/g ww). Age had a positive effect on the liver concentrations of metals, while no effect was found for sex or histological changes. The prevalence of histological changes in liver tissue decreased in the following order: random pattern mononuclear cell infiltration (92.1%), portal cell infiltration (68.4%), hepatic intracellular fat (18.4%), portal fibrosis (7.9%), focal hepatic fibrosis (7.9%), bile duct hyperplasia/fibrosis (7.9%) and lipid granuloma (2.6%). For kidney tissue, the prevalence of histological changes decreased in the following order: glomerular mesangial deposits (54.1%) > glomerular basement membrane thickening (45.9%) > THD (40%) > tubular hyaline casts (14.0%) > glomerular atrophy (13.5%) > dilated tubules (13.5%) > glomerular hyper-cellularity (10.8%) > mononuclear cell infiltrations (8.1%).

## 1. Introduction

Biota and local communities of Greenland and the circumpolar area in general are influenced by the anthropogenic contamination of water, soil and air largely by persistent organic pollutants (POPs) and heavy metals such as cadmium (Cd) and mercury (Hg) [1,2,3,4,5,6,7,8,9,10,11,12]. Up to 95% of the mercury pollution originates from industrialized areas at lower industrialized latitudes where to it is deposited via long-range atmospheric and sea current transport [13,14]. It has been shown that several biological systems have the capacity to accumulate these contaminants to degrees raising general concern for the sustainability of a healthy ecosystem [12,13]. Thus far, there has been convincing evidence that environmental contaminants can cause wildlife mortality directly/indirectly by suppressing immune function, altering behavior, reducing fertility and generally reducing the body condition score in challenged wildlife [15,16]. In the case of Hg, this metal has been seen to increase during the last decade in Greenland and the Canadian Arctic, while Cd has been found to be somewhat steady [17].

Top trophic predators like polar bears (*Ursus maritimus*), killer whales (*Orcinus orca*) and ringed seals (*Pusa hispida*) accumulate the highest heavy metal concentrations including Cd and Hg in the Arctic marine environment and this accumulation increases with age [1,2,14,17,18,19,20,21]. Opposite to terrestrial mammals, marine mammals are exposed to high levels of Hg and Cd due to natural background concentrations from the bedrock. Therefore, these species have evolved various detoxification mechanisms including insoluble mercuric selenide (tiemannite) HgSe complex deposition in the liver, as well as metallothionein Cd-binding in various tissues including the liver and kidney [21,22]. Indeed, it has been estimated that after a 150-year continuous Hg increase, it has come to the point where more than 90% of the Hg body burden of higher trophic species such as the ringed seal stems from anthropogenic Hg [8,14]. The Greenland ringed seals play an important role for the local Inuits who still regularly hunt and consume this species, thereby rendering themselves vulnerable to the same contamination-related risks as the seals may be challenged with [1,2]. This point and the facts that ringed seals are geographically widespread, easy to collect, sensitive to change and bio-accumulate environmental pollution make them ideal as a biomonitoring species in the Arctic Monitoring and Assessment Program (AMAP) [2]. The issue of humans acquiring considerable amounts of heavy metals or other environmental contaminants through a marine mammal diet is of concern for people in Greenland and other Arctic areas [23]. However, it is recognized as a worldwide problem and has been coined: “the global, silent pandemic of neurodevelopmental toxicity” [6]. In particular, methylmercury (MHg) is, among other characteristics, a known developmental neurotoxicant [24,25,26,27] and is generally the Hg compound of most concern in terms of human health. This is partly because the percent contribution of MHg to total mercury (THg) is high in muscle (edible tissue) [28,29], is readily absorbed from the food and has the ability to cross the blood–brain barrier where it causes some of its characteristic neurotoxic effects [30,31,32].

Cd and Hg toxicity share some important similarities: They are toxic to several biological systems and their mode of toxicity is, among others, greatly mediated through increased oxidative stress by the generation of reactive oxygen species (ROS) since they are antioxidant selenium (Se) scavengers [33]. Cd has, moreover, a strong affinity for SH groups, and while many essential enzymes consist of such entities, Cd acts as an enzymatic inhibitor [34,35]. The kidney is considered the primary organ for Cd toxicosis, but in experimental studies with rats fed low dose/long-term Cd, liver lesions preceded kidney lesions [36,37]. Other than the ability to cause a relatively large calcium deficiency and thereby lead to osteoporosis [38,39], cadmium is also known to be a carcinogenic in at least terrestrial animals [40]. It should be noted, however, that the pathological effects generated from the exposure of terrestrial mammals to Cd do not necessarily apply to predator marine mammals, as evolution seems to play a large role compared to metallothionein Cd-binding as stated further above [41].

Mercury originates from both natural and anthropogenic sources [42] and exists in different forms, all exerting an array of different toxicities including immunosuppression, reduced fertility, endocrine disruptions and an increased mortality rate [15,16]. As already mentioned, one of the toxicity modes of Hg is through increasing oxidative stress. Hg has strong affinity for Se, and while many of the antioxidative enzymes responsible for protection against oxidative stress consist of a Se core, these enzymes can be attenuated by Hg, thereby increasing the damaging effects of free radicals [40,43,44]. Because of Hg’s strong affinity for Se, this can, on the other hand, also render a detoxification and sequestration of Hg by binding into the insoluble tiemannite complex [45,46]. Thus, even though we still do not know what the toxic threshold level is for Hg in marine mammals, it is evident that such a threshold should be seen in the light of concurrent Se levels. Still, it is expectable that there is an upper limit to the capacity of Se detoxification and precipitation of the resistant tiemannite crystals—that the protective capacity of Se is, in other words, not limitless.

In spite of considerable amounts of conducted surveys monitoring the levels of non-essential heavy metals in arctic wildlife, still very little is known of the chronic effects and population health consequences of relatively high heavy metal levels in these animals—not least concerning marine mammals. One of the reasons for this is that there still is a general lack of concurrent ADME (Absorption, Distribution, Metabolism, Excretion) studies and clinical–pathological parameters analyzed in relation to heavy metal burden in wildlife [42]. Based on this, we therefore decided to evaluate Cd, Hg and Se levels in relation to histological changes in 40 West Greenland ringed seals collected in Qeqertarsuaq (Godhavn) and Qaanaaq (Thule), 2008.

## 2. Materials and Methods

### 2.1. Sampling and Age Estimation

**Figure 1:**
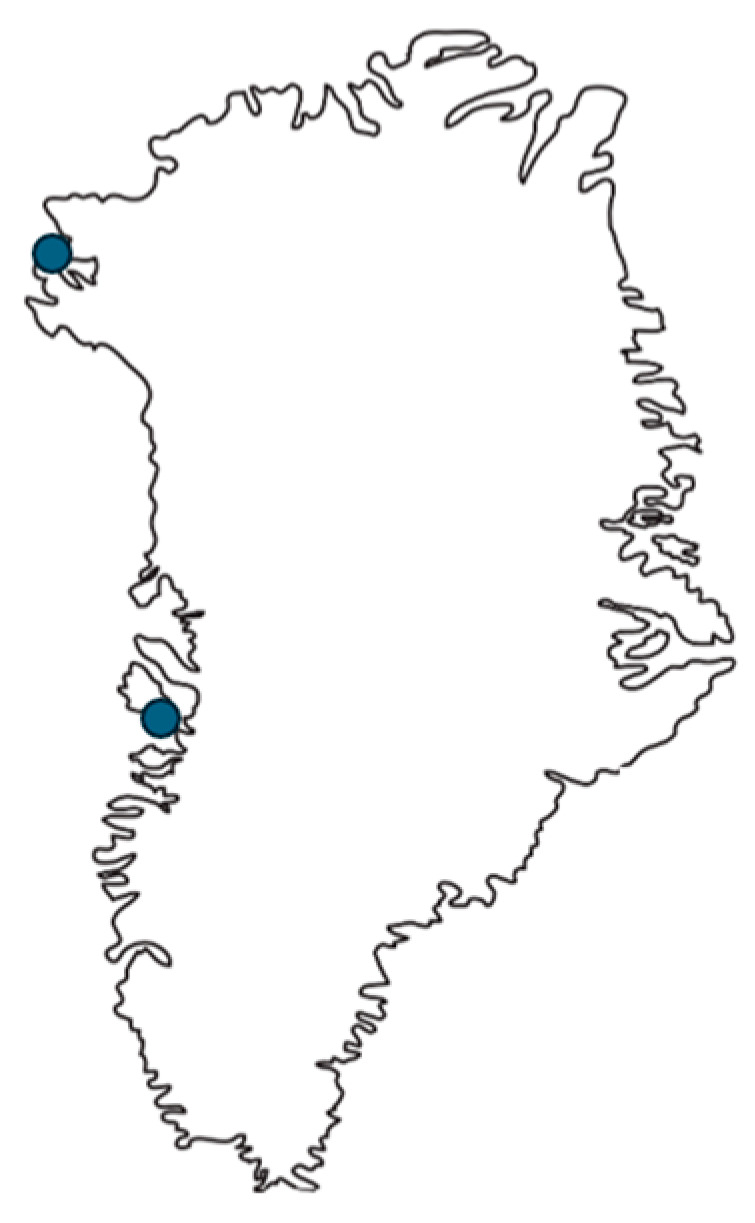
The location of Qaanaaq in North West Greenland and Qeqertarsuaq in Central West Greenland shown by blue circles.

Samples were collected from ringed seals in the Qaanaaq (*n* = 20) and Qeqertarsuaq (*n* = 20) areas in Greenland (Figure 1), May 2008, as part of the annual subsistence hunting allowing access to a limited number of individual animals that could be sampled. The samples were taken within 1–12 h post mortem as part of local subsistence hunting (decomposition code 1). Due to logistical difficulties, it was not possible to obtain formaldehyde-fixed tissue from all individuals. The insulating effect of the blubber assisted to counteract freezing of the internal organs prior to sampling. Samples from the kidney and liver were taken from each seal and stored in separate PE plastic bags. Small fragments of the kidney (ca. 2 cm × 2 cm × 2 cm) were stored in an antifreeze fixative containing 10% of formalin (35% formaldehyde) solution and 90% of ethanol (96%) to prevent tissue samples from being subjected to freeze damage. Samples were kept at the outdoor temperature (−5 to −20 °C) for up to five days pending controlled freeze storage at −20 °C. Age estimation from cementum Growth Layer Groups (GLGs) of the lower left canine was carried out by the Canadian Wildlife Service in Edmonton, Canada, and at the trace element laboratory at the Department of Ecoscience (ECOS), Aarhus University in Roskilde, Denmark, following the method described by Dietz and coworkers [47].

### 2.2. Metal Analyses

Liver tissue was analysed for Se, Hg and Cd at the trace element laboratory at ECOS, Aarhus University in Roskilde, Denmark. Sub-samples of liver (≈1000 mg wet wt.) were cut out from the liver using a stainless-steel scalpel. All surfaces of the sub-samples were freshly cut to avoid contamination. Sub-samples were weighted and microwave digested in Teflon bombs in 4 mL/4 mL Merck Suprapure HNO_3_/milliQ water using an Anton Paar Multiwave 3000 microwave oven (Anton Paar, Graz, Austria). Digestion solutions were diluted with milliQ water, and Se and Hg were determined in the solutions following a reduction with sodium borohydride in a flow injection system. A Perkin Elmer Flow Injection Mercury System (FIMS) (Perkin Elmer, Waltham, MA, USA) was used to determine concentrations of Hg, while Se was determined coupling the FIMS to a Flame Atomic Absorption Spectroscopy instrument (Perkin Elmer AAnalyst 300, Perkin Elmer, Waltham, MA, USA) with a heated quartz tube mounted in the light path. Cd was determined using Graphite Furnace or Flame Atomic Absorption Spectroscopy (Perkin Elmer Zeeman AAnalyst 800 or Perkin Elmer AAnalyst 300 (both Perkin Elmer, Waltham, MA, USA), respectively). The laboratory at BIOS was accredited for the analyses of Se, Hg and Cd in biota with 10%, 12.5% and 25% precision (2 SD), respectively. Accredited detection limits for Se, Hg and Cd were 0.2, 0.005 and 0.002 mg kg^−1^ dry wt. The analytical quality of the chemical analyses was checked by analyzing blanks, duplicates and the certified reference materials along with the samples. The certified reference materials DOLT-3 and DORM-2 (fish liver and fish protein) from the National Research Council Canada were used and the recoveries of Se, Hg and Cd in the certified reference materials were 92–107% during the analyses. All concentrations are given on a wet weight (ww) basis. Molar Hg–Se ratios were calculated using atomic weights for Hg (200.59 g/mol) and Se (78.96 g/mol).

### 2.3. Histology

Tissue was trimmed, processed conventionally, embedded in paraffin, cut into 4 µm sections and stained with Haematoxylin (Al-Haematein)–Eosin (HE) and Periodic acid–Schiff (PAS) to emphasize basement membranes and connective tissue, and, thereby, glomerular (capillary and mesangial), tubular and interstitial changes [48,49,50]. All slides were evaluated in low (40×) to high (600×) power fields with an Olympus BX60 microscope (Olympus, Tokyo, Japan) with an added Leica DC500 camera (Leica, Wetzlar, Germany). Eight hepatic changes were recorded: mononuclear infiltration (MCI), portal cell infiltration (PCI), portal fibrosis (PF), focal hepatic fibrosis (FHF), hemosiderosis (hem.), bile duct hyperplasia/fibrosis (BHF), lipid granuloma (LG), hepatic intracellular fat (HIF) and Ito/stellate cell abundance (ITO), and 11 renal changes: glomerular basement membrane thickening (GBMT), glomerular atrophy (GA), glomerular mesangial deposits (GMD), glomerular hyper-cellularity (GHC), dilated tubules (DT), tubular hyaline droplets (THD), mononuclear cell infiltration (MCI), tubular hyaline casts (THC), glomerular sclerosis (GS), interstitial fibrosis (IF) and tubular necrosis (TN). Each histologic change was scored on a four-step scale: (1) non-appearing, (2) mild degree, (3) moderate degree and (4) severe degree. The respective criteria for each of these scores are given in Table 1. Slides were evaluated twice and blinded. Results did not differ significantly between trials. To diagnose hemosiderosis, suspected specimens were stained with an iron stain, more specifically, Perl’s stain, which depends on a Prussian blue reaction [50,51]. To differentiate between intracellular lipid and glycogen, relevant specimens were PAS stained to accentuate the appearance of glycogen.

### 2.4. Statistics

The statistical analyses were performed with the SAS statistical software package (SAS V9 and Enterprise Guide V4, SAS Institute, Cary, NC, USA) and the free software R version 3.01 (R Core Team, 2013). The level of significance was set at *p* ≤ 0.05, while 0.05 < *p* < 0.1 was considered a trend. Student’s t-test was used to test the relationship between age and metal. Finally, Chi-square tests were performed to test for histopathology prevalence between age/sex groups. A general linear model (GLM) was applied to test the relationships between histopathology prevalence and age and metal concentration. Age was square-root transformed and metal concentrations were log-transformed (base e) prior to the analyses in order to approach the assumption of normality and homogeneity of the variance [52]. A binomial error structure of the GLM analyses was applied in the case of present versus not present data and a Poisson error structure in the case of score data. In the following, x ± y will be used to denote the arithmetic mean ± the standard deviation.

## 3. Results

### 3.1. Age and Sex Distribution

All animals were freshly sampled, and tissues were immediately fixed in formaldehyde for histology and frozen for chemical analyses at minus 20 °C. Females were slightly overrepresented in the Qaanaaq (12/20) area, while males were slightly overrepresented in the Qeqertarsuaq (13/20) area of Greenland. Regarding age, there was a general tendency of catching only sub-adult seals a max of 3 years (0–3 years) old in Qeqertarsuaq, whereas 2/20 (10%) of the seals caught in Qaanaaq were adults (8 and 11 years old, respectively), while the rest were 0–3 years old.

### 3.2. Metals

Results from the element analyses are shown in Table 2. The highest Hg concentration of 23.29 µg/g ww was found in an 11-year-old female seal collected in Qaanaaq. The mean Hg concentration for Qaanaaq was 1.738 ± 1.70 µg/g ww, while for Qeqertarsuaq, it was 1.78 ± 1.70 µg/g ww (Table 2). For Cd, the highest concentration was found in an 8-year-old female from Qaanaaq with 38.79 µg/g ww. The mean Cd for Qaanaaq was 7.80 ± 8.95 µg/g ww, while it was 11.58 ± 6.32 for Qeqertarsuaq (Table 2). The overall mean Hg–Se ratio was 0.45 ± 0.19 and only exceeded 1 for a single female in Qaanaaq (Se–Hg = 1.13), which may indicate increased risk for oxidative stress (Table 2).

### 3.3. Liver Histology

**Figure 2:**
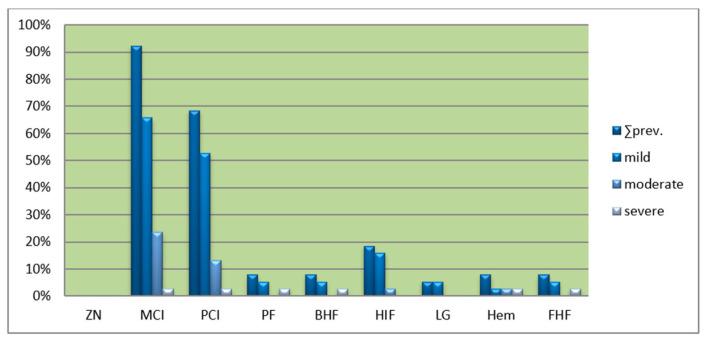
Prevalences of liver changes in the ringed seals sampled in Qeqertarsuaq and Qaanaaq areas of Greenland in 2008. ZN = zonal necrosis, MCI = mononuclear cell infiltration, PCI = portal cell infiltration, PF = portal fibrosis, BHF = bile duct hyperplasia/fibrosis, HIF = hepatic intracellular fat, LG = lipid granuloma, Hem = hemosiderosis, FHF = Focal hepatic fibrosis.

**Figure 3:**
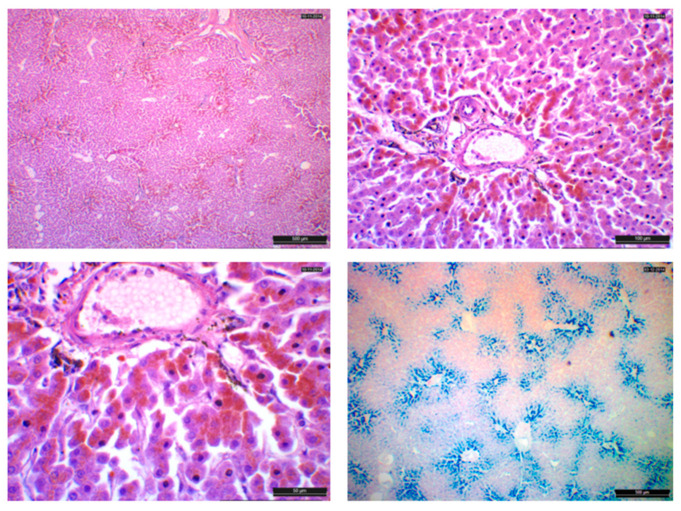
Hemosiderin shown at three magnifications. **Top left**: portal hemosiderin. 40xHPF, bar scale = 500 µm; **top right**: portal hemosiderin 200xHPF, bar scale = 100 µm; **bottom left**: portal hemosiderin 400xHPF, bar scale = 50 µm; **bottom right**: Perl’s stain, hemosiderin staining blue, 4xHPF, bar scale = 500 µm.

The prevalences of histological changes in the liver tissue of the seals from Qaanaaq and Qeqertarsuaq are shown in Figure 2. It is seen that portal and parenchymal mononuclear cell infiltrations are found in most of the seals, while the remaining six changes are only found in 2–20% of the specimens. The overall specific liver change percentages for both Qeqertarsuaq and Qaanaaq were: MCI: 92.1%, PCI: 68.4%, HIF: 18.4%, PF: 7.9%, Hem: 7.9%, FHF: 7.9%, BHF: 7.9% and LG: 2.6% (Figure 2). Portal hemosiderosis was apparent in three liver slides, with one of them grouped as being severe (Figure 3).

It should be noted that bodies of the liver fluke (*Orthosplanchnus arcticus*) were with certainty found in the cross-sections of three seals sampled in Qaanaaq in 2008. The histologic changes in at least two of these did indeed coincide with the pathognomonic histopathological changes of this parasite including portal and hepatic fibrosis and bile duct hyperplasia and fibrosis.

### 3.4. Kidney Histology

**Figure 4:**
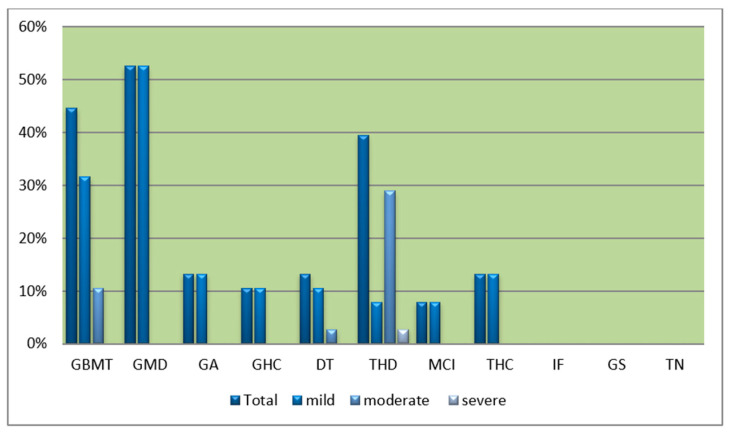
Prevalences of histological changes in kidney tissue from 38 ringed seals sampled in Qeqertarsuaq and Qaanaaq areas of Greenland.

**Figure 5:**
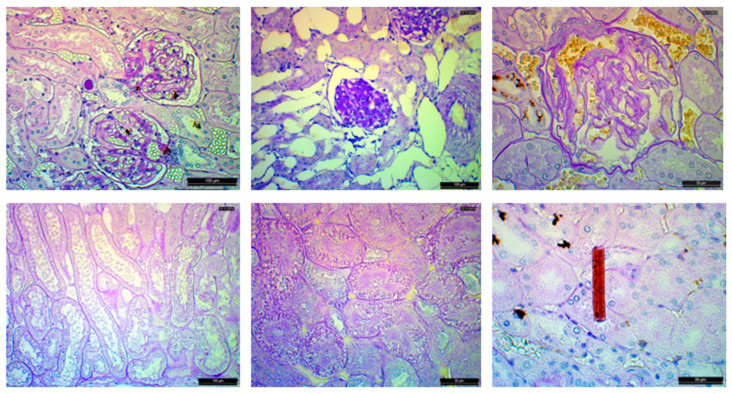
Examples of recorded histologic changes. **Top left**: two normal ringed seal (*Pusa hispida*) glomeruli, bar scale: 100 µm; **top center**: a glomerulus with hyper-cellularity and increased mesangial matrix, bar scale: 100 µm; **top right**: glomerulus with moderate sclerosis of the visceral and parietal basement membrane and mesangial deposits, bar scale: 50 µm; **bottom left**: dilated tubules, bar scale 100 µm; **bottom center**: hyaline droplets probably originating from reabsorbed protein in proximal tubules, bar scale: 50 µm; **bottom right**: presumed struvite crystal, bar scale: 50 µm.

The frequency and severity of renal histological changes are shown in Figure 4. It is seen that glomerular atrophy/sclerosis, when present, was only presented by a few atrophic/sclerotic glomeruli. No tubular necrosis was observed and none of the kidney specimens displayed suggestions of renal failure. A presumed crystalluric struvite (coffin-lid shape) crystal was found in one histologic preparation (Figure 5).

### 3.5. Histology vs. Heavy Metals, Selenium and Age

Age had a positive effect on all element concentrations (all *p* < 0.04), while there was no effect from sex (all *p* > 0.05). There was, however, an effect from location with Qaanaaq seals having the highest liver concentrations of elements (all *p* < 0.05). No differences was found between sex and any of the heavy metals (Cd Hg) and selenium, and therefore, sex was not included in further statistical analyses.

Results from the GLM analyses are found in Table 3 and Table 4. It is seen that there was an effect from age on PCI, PF, BHF and FHF. Likewise, there was an effect from Hg, Cd and Se on hemosiderosis and Cd on PF. For histological changes in kidney, age had an effect on GHC, DT, THC and GS.

## 4. Discussion

The current study is part of the Arctic Monitoring and Assessment Program that is part of the Arctic Council with the overall aim to reduce emissions and the long-range transport of contaminants into the Arctic to sustain biodiversity and human health.

### 4.1. Heavy Metals

Hg preferably accumulates in the liver, whereas Cd concentrates in the kidneys [18,28,53,54]. Koeman and coworkers [55] ultra-centrifuged various tissues from one Hg-contaminated *Phoca vitulina* (harbor seal) and found 55% of the Hg to be present in the liver, 46% in the kidneys and 34% in the brain. Generally, the Cd and Hg liver levels of the ringed seals of the present study were much elevated compared to what are considered typical levels in domestic species, but they were still within ranges previously reported for arctic marine mammals [1,18,53,54,56]. Wagemann and coworkers [53] conducted an extensive survey on heavy metals in Canadian Arctic marine mammals from the period 1978–1994 and, over this period, reported mean Hg values from 284 ringed seals, age 0–38 years old, that ranged from 0.23 µg/g ww to 219 µg/g ww in the liver. A significant difference was also found for generally higher Hg levels in Western Arctic compared to Eastern Arctic ringed seals. This trend was, though, not apparent for Cd where no significant difference could be found between Eastern and Western Arctic seals. The mean Cd in the livers of the ringed seals ranged from <0.002 to 44.6 µg/g ww, while Se ranged from 0.51 to 65.3 µg/g ww in the liver. Because the range of the recordings during this extensive survey encompasses values from one extreme to the other, it is not surprising that our results fall within these ranges.

Aubail and coworkers [57] reported the results of these consecutive samplings, revealing an overall Hg mean and range for these 115 animals that corresponds with the findings in this study by a mean of 2.94 ± 1.99 µg/g ww and range of 0.43–10.99 µg/g ww. They also sampled 24 ringed seals from the vicinity of Qaanaaq sometime in the rather wide time span 1996–2006 (no further time specifications given) and found a mean Hg of 3.06 ± 0.49 and range of 0.87–10.28, which additionally corresponds neatly with both the present results and the results given by Aubail and coworkers [57]. Further such studies of Hg in the liver tissue of ringed seals from around Qaanaaq and Qeqertarsuaq have been conducted previously [17] and the results of the present study fell in line with these. For liver Cd concentration, to our knowledge, only one other study has reported such results from either Qaanaaq or Qeqertarsuaq. In this case, it is a report from Qeqertarsuaq, *n* = 25; 1994–1995, revealing a mean liver Cd concentration of 19.48 ± 7.49 µg/g ww with no range given [17]. This is a considerably higher mean than that reported in the present study (11.58 ± 6.32 µg/g ww), but the former study also comprised significantly older animals with 56% being older than 4 years compared to none above 4 years in the present study. While the concentration is strongly age-related, this difference between our two surveys is not overwhelming.

Hg and Cd in liver tissues increased with age in the present study, which is in accordance with the literature [18,41,53,54,58,59,60,61]. The oldest seal in the present study had the absolute highest Hg–Se of 1.13. The Hg–Se ratio is a comprehensive criterion for proposing and assessing health risks associated with Hg toxicity. It is not fully understood how this protection unfolds, but it may be through a sequestration of Hg because of a very high binding affinity between Hg and Se. Thereby, Se prevents Hg attenuation of free radical sequestering Se-rich enzymes, as well as sustaining the production of these Se-rich enzymes by its availability [62,63]. From a theoretic basis, the Se-Hg sequestering hypothesis is widely accepted as a protection against Hg adverse toxic effects when the Hg–Se molar is below 1 [64,65,66,67]. A rat study by Ralston and coworkers showed that the Hg–Se ratio was as high as 14 in rats fed high methyl-Hg/low Se diets, whereas this ratio was only 4 in rats fed a high methyl-Hg/high Se diet [67]. From this perspective, the majority of the seals of this study were not at immediate risk of Hg toxicity from unsequestered organic and inorganic Hg as all but one had Hg–Se ratios below 1.

The mentioned oldest seal in this study with the highest held Hg liver concentration had a very modest Cd concentration of 3.65 µg/g ww, while the second oldest seal held the top Cd liver concentration of 38.79 µg/g ww. It should be taken into consideration here that sufficiently damaged kidneys can begin to leak Cd and thereby yield a lower concentration. If the Cd concentration in the body acted as equilibrium so that Cd would redistribute from the liver in response to such a Cd renal leak, then that could explain the relatively low Cd concentration in this older and otherwise metal-burdened seal [41]. Accordingly, considerable pathologic lesions were found in this animal (GBMT, GA, GHC, GMD and DT). It remains unknown if these lesions were sufficient to cause a Cd leak.

### 4.2. Liver Histopathology

The etiologies for the liver histological changes can be anything that has the potential to focally damage the liver tissue such as pathogens: parasites, bacteria, virus and fungi. Moreover, contaminants may play a role in the development of these lesions—either directly or indirectly via immune suppression [15,16]. Since more than 90% of the samples showed various degrees of focal mononuclear cell infiltration, this could be—to a certain extent—considered within the normo-physiological range in response to inevitable low-virulence pathogens or other mildly tissue-damaging agents. Generalized liver changes were only found in 2 of the 40 seals, both from Qaanaaq and adult seals. These changes were generalized hepatic fibrosis and bile duct hyperplasia. These two specimens were found to be concurrently infested with the liver fluke *O. arcticus* [68].

Sonne and coworkers screened liver and kidney histopathology from long-finned pilot whales (*Globicephala melas*) from the Faroe Islands and narwhals (*Monodon monoceros*) from Northwest Greenland in relation to Hg and Cd [11,54]. These animals had higher levels of Cd and Hg than the ringed seals of this study; specifically, 50 times higher mean liver Hg and 1.5 times higher mean Cd. Many of the same lesions as in this study were recorded in both studies, yet most with a higher frequency. In fact, for the pilot whales, all of the seven lesions recorded displayed a frequency of 30% or above (*n* = 14, all adult except one juvenile). The highest scorers were portal cell infiltration (64%), followed by focal necrosis (57%) and intra-hepatocellular lipids (50%). In our study, focal necrosis in the form of focal mononuclear cell infiltration, portal cell infiltration and intra-hepatocellular lipids were also the exact same top three scorers in the sense of prevalence. For the narwhals from Northwest Greenland (*n* = 12, all adult except two sub-adults), yet again, the lesions recorded here were replicated in the present study, and here, portal cell infiltration and intra-hepatocellular lipids were also among the top three liver lesions recorded, both with prevalences above 25%. Generally, lesions revealed lower frequencies in this study than those related to the pilot whales, with all pathologic lesions showing frequencies of less than 40%. Only Hg was measured in these narwhals and at a level 11 times lower than that for the pilot whales: 11.88 ± 10.47. Woshner and coworkers [69] measured selected elements including Hg and Cd in 55 Alaskan bowhead whales (*Balaena mysticetus*) (1983–1990; 1995–1997), beluga whale (*Delphinapterus leucas*) (1992–1995; 1996–1997) and ringed seals (1996–1997) from the Canadian Arctic, and the histology of the liver and kidney wass also evaluated. For the bowhead whales (*n* = 55) with a low Hg liver mean of 0.06 ± 0.073 µg/g ww and Cd mean of 9.63 ± 10.43 µg/g ww, no pathologic changes were reported, with only moderate amounts of the typical “wear-and-tear/age” pigment lipofuscin around bile canaliculi and rare accounts of hemosiderin. In beluga whales (*n* = 24) with a much higher mean liver Hg of 82.47 ± 25.93 µg/g ww and Cd (*n* = 50) mean of 3.81 ± 1.76 µg/g ww, portal cell infiltration and fibrosis were found, with moderate amounts of lipofuscin and, rarely, hemosiderin otherwise abundant in Ito cells. Portal cell infiltration was also frequently found in the present investigation. On the other hand, we only rarely found lipofuscin, but found two seals with moderate hemosiderosis, one with a mild case, and found that, for all, the hemosiderin was not isolated to the Ito cells but concentrated around portal areas. This latter picture is confirmed in Woshner’s ringed seals where hemosiderin was also found and seen to be intra-hepatocellular (no. of affected seals not given). These ringed seals had a Hg liver (*n* = 16) mean comparable to our study: 3.52 ± 5.07 µg/g ww, and a Cd liver (*n* = 17) mean of 5.72 ± 3.21 µg/g ww. The lesions Woshner (2000) recorded are in accordance with our study material: in 9/15 seals, mild to moderate multifocal and/or portal cell infiltration (hepatitis) and necrosis was found. In one seal otherwise apparently devoid of lesions, moderate hemosiderosis was found in the before-mentioned pattern. A comparison to metal levels in the given seal was not presented.

Even though some lesions are replicated between studies of animals with relatively high metal levels, because these frequent lesions are unspecific and to some degree also found in healthy animals, it cannot be concluded that they are directly or indirectly caused by heavy metals. Still, both in vivo and in vitro experiments have shown immunosuppressant and endocrine disruptive traits [70,71,72], and that these disruptions may occur even without light microscopic lesions. It is not unlikely that these effects are ongoing in the seals or additionally that they precipitate the lesions found. These lesions are otherwise mostly signs of various disease processes caused by pathogens like bacteria, virus, parasites and/or pathogenic fungi. However, in rats chronically exposed to low levels of Cd (0.5 mg/kg 6 days/week for up to 26 weeks), hepatic injury was found to precede nephrotoxicity and one of the concurrent histological features was focal hepatic necrosis, and later interstitial fibrosis in all zones of the liver lobule [37]. Small areas of focal necrosis observed as focal mononuclear cell infiltration were frequently found in the present study.

### 4.3. Kidney Histopathology

Sonne and coworkers evaluated kidney lesions in relation to a mean 44.5 ± 40.8 µg/g ww Cd concentration in 100 ringed seals from Qaanaaq from 1998 [73]. From AMAP [1]-compiled data from 184 ringed seals, it is seen that the Cd kidney concentrations are between 2 and 4 times higher than those of the liver. The same relationship was seen in 16 minke whales off the coast of West Greenland [57]. Taking this into account, the Sonne study’s [73] mean concentration is highly comparable with the Cd mean concentrations for Qeqertarsuaq of 11.58 ± 6.32 and Qaanaaq of 7.80 ± 8.95 µg/g ww. The lesions found in [73] were mesangial deposits, interstitial nephritis, glomerulonephritis and arteriosclerosis, but they were only found in 10/100 seals and could not be attributed to Cd directly. Characteristically, this is to some degree in agreement with our histological findings as we also found a low prevalence of these lesions (glomerulonephritis and arteriosclerosis in the sense of mononuclear cell infiltration and/or thickened glomerular basement membranes). Sonne and coworkers [11,54] also looked at the kidney histology in Hg- and Cd-screened narwhals and pilot whales that, in the case of the pilot whales, displayed significantly higher heavy metal contamination. The kidney lesions studied in these two investigations were characteristically close to those presented here, yet with higher prevalences: seven out of eight lesions were above 20%, ranging from 21 to 71% for the pilot whales. The latter prevalence was presented by dilatation/hyalinization of Bowman’s capsule, a lesion not recorded in this study. For the narwhals, six out of seven lesions were above 20% prevalence ranging from 33% to 100%, the latter suggesting that assumed glomerular capillary membrane thickening may be a normal variation in this species. Comparatively, as for the top three recorded renal lesions of this study, the equivalent recordings in the narwhal and pilot whales were accounted for at frequencies between 20 and 40 percent (except for the narwhal 100% prevalence basement membrane thickening account).

Thickened Bowman’s capsules (14/17) and increased mesangial deposits (6/14) were also reported by Woshner and coworkers [69] in beluga whales with prevalences comparable to the ringed seals of this study. They also found suggestive evidence that increased mesangial deposits could be linked to Hg and Cd levels. In addition, they observed mild renal lithiasis in 7/17 beluga whales, for which it is noteworthy that we found a crystal, analogous to struvite crystals, in one of the renal histology samples, and renal lithiasis was also found in 4 of the 12 ringed seals reported by Woshner and coworkers [69]. Tubular necrosis—a typical finding in Cd acute/sub-acute toxicosis—was not found in either of the studies, including the present one. Mercury can also induce tubular necrosis and the basement membrane is preserved [74]. This facilitates regeneration of the tubular epithelium with the remaining viable cells spreading to cover exposed portions of the basement membrane, lending a flattened appearance to the tubular epithelium. The dilated tubules observed in this study could have been a result of this, while flat tubular cells were found in conjunction.

The etiology of glomerular (and capsular) basement membrane thickening is most prominently immune-mediated glomerulonephritis. It is caused by the deposition of soluble immune complexes in the basement membrane, subendothelially or subepithelially triggering local inflammation and, hence, a thickened basement membrane and increased mesangial matrix [75]. It can be driven by long-term chronic infections resulting in hyperglobulinemia (and its antigen–antibody complexes) and less commonly by specific antibodies targeting particular corpuscular structures (autoimmunity). There are at least two ways Cd and Hg could influence this pathogenesis: by the immune disrupting properties (direct and indirectly via endocrine effects) of the heavy metals either triggering autoimmunity or reducing immunocompetence towards infections creating more persistent infections. However, it is difficult to ascribe the findings to heavy metals in wild marine mammals as long as control groups cannot be established. However, in controlled rat experiments, Hg administration induced glomerulonephritis and increased mesangial deposits apparently through immune-mediated mechanisms [76,77]. These features were also observed relatively frequently here. The intracellular hyaline droplets seen in the proximal tubular cells were most likely reabsorbed protein from the ultrafiltrate in the form of eosinophilic proteinaceous droplets. The etiology of this is believed to be anything that increases the protein amount in the ultrafiltrate such as protein-losing nephropathies, one being glomerulonephritis, but also tubular necrosis [78]. Among terrestrial mammals, including humans, chronic Cd toxicosis is among other clinical signs seen as a low molecular weight proteinuria. In these species, a renal cortical concentration of 200 µg/g ww has been set as the critical threshold for chronic Cd toxicosis [79]. This concentration is not exceeded by our liver Cd levels even if the kidney concentration can be expected to be four times higher.

### 4.4. Histopathology vs. Metals and Age

Se and Hg were highly correlated to age, in concordance with previous studies. Cd was also found to be positively correlated to age, yet not as strongly as Hg. For the liver, a positive correlation was found between age and portal fibrosis, focal hepatic fibrosis, portal cell infiltration and bile duct proliferation. This is not a surprising result as these changes tend to be age related [11,54,80], and in the case of bile duct proliferation and hepatic fibrosis, it was observed that the two oldest individuals were infested with a liver fluke, which initiates this lesion, and thus were responsible for much of the significance of the result. The changes that were observed across studies (mentioned above) and species with considerable heavy metal contamination: focal cell infiltration, portal cell infiltration and portal fibrosis, were, in this study, not found to correlate significantly with either of the elements, and it therefore offers the suggestion that these lesions are generally age related; accordingly, as mentioned above, two of these lesions yielded a statistically significant correlation to age.

The characteristics behind the statistically suggested link between Hg, Cd, Se and hemosiderosis are not known. Hemosiderosis is thought to appear when the liver’s capacity to metabolize excess iron is saturated and the ferritin iron complexes accumulate in hepatocytes [81]. This can either be because of the decreased capacity of the liver or increased load/demand, for example, as a result of a hemolytic crisis. While the effect from metal concentrations on hemosiderosis was negative, it could be speculated if some unknown factor, likewise linked to the increased accumulation of hemosiderin, caused the hepatocytes to “leak” heavy metal, and thus, paired blood and urine analyses would be preferable in future equivalent studies. Because of the characteristics of the metal quantification method, it is not presumable that metal has been bound in complexes of the hemosiderin-like accumulations, making it unavailable for analysis. The analytical methods here are indiscriminate of that and find the total Hg and/or Cd.

In relation to the kidney lesions, none of these were found to be statistically correlated to any of the elements. All correlations were in relation to age. When location was included in the analysis, glomerular basement membrane thickening was strongly correlated to it, but the quality of this result was speculative as there were only four recordings of glomerular basement membrane thickening all together. If, however, it represents a true picture, it is correspondingly noteworthy that in spite of the generally younger age of the Qeqertarsuaq seals, the mean Cd levels were higher in these animals than in the older Qaanaaq sample. Thus, with some skepticism still retained, this could be an indication of the Cd-induced histopathology of the ringed seal by long-term exposure, on the basis of: it is the younger group (Qeqertarsuaq) that reveals a higher frequency of the lesion glomerular basement membrane thickening, and this renal lesion has been reported in controlled laboratory studies of cadmium dosing.

As already mentioned, it is difficult to make a conclusion about the effects of chronic heavy metal challenges in wild species when control groups are difficult to obtain. The changes seen here for the kidney and liver could solely be due to age and a normal exposure to different pathogenic agents and physio-developmental challenges such as acquired autoimmunity. However, as these metals have experimentally displayed immune suppressant effects, they could be chronically accentuating all the before mentioned challenges. There is a clear need for controlled studies, but when these are not possible, statistical correlation analyses can sometimes provide some insight. We will proceed with this in the following studies.

### 4.5. Implications

Relative to what is stated above, the population health implications for the ringed seal are difficult to assess without further studies including histopathology or other modalities used in conjunction with heavy metal quantification. As it has not yet been possible to set a satisfactory threshold for Cd or Hg levels in any marine mammal, we cannot evaluate the given metal results in this light. However, recently, suggestions were found of an increased liver fluke burden (also found in the seals of this study) in the seals sampled by the AMAP initiative. It is possible that the current heavy metal and/or POP burden is driving this suggestive increased parasite prevalence. Moreover, if the seals are truly experiencing decreased immunocompetence, they can potentially harbor more pathogens including those with a zoonotic potential, thereby increasing the risk of human health implications. It is noteworthy that some local Inuit groups still consume parts of the animals raw. Already, the local Inuit people’s health is threatened by the high amounts of heavy metals (and POPs) [1,82,83,84] accumulated in their traditional game, which many of these people still rely on as a significant food source. In respect to the given guidelines of WHO, there is too much Hg in these animals to warrant them as a significant food source for humans. Marine mammals seem to display an unusual tolerance toward heavy metal burdens, partly through the protective mechanisms of Se in the case of Hg forcing a Se evaluation in conjunction with metal quantification. Extrapolation from toxicological studies of terrestrial animals should, in the regard of estimating health impacts, be performed with extreme caution. Nevertheless, it seems inevitable that there is an upper limit to the protective capacity of Se against Hg (and vice versa) [41].

## 5. Conclusions

A wide array of mild to moderate degree liver and renal lesions was found across age groups of ringed seals. The specific changes found were compatible with other contaminant-concerning histologic studies of heavily contaminated marine mammals. Only portal fibrosis and hemosiderosis were linked to Hg or Cd. While little is known about the long-term chronic effects of these metals, except for experimental studies revealing endocrine and immunosuppressant effects, and because the mode of toxicity and mode of general ageing share similar characteristics (oxidative stress), we cannot reject the likelihood of heavy metals precipitating various disease processes and organ lesions in affected ringed seals. This is an important result for the monitoring of the circumpolar environment for long-range transported pollution including mercury as it can be used by the United Nations Environment Programme to reduce industrial emissions and the adverse effects on biodiversity and human health.

## Figures and Tables

**Table 1:** Overview of the criteria for scoring for each histologic change in liver and kidney. Four histological scores: not found, mild, moderate and severe. Sum: sum of which the lesion/change was found per 40xhigh-power field (HPF), * = per 200xHPF.

**Liver**			
Histopathological change	Mild	Moderate	Severe
MCI = mononuclear cell infiltration	sum < 9	9 ≤ sum < 20	sum ≥ 20
PCI = portal cell infiltration (PA = portal area)	≤25% of PAs	≤50% of PAs	>50% of Pas
PF = portal fibrosis	≤25% of PAs	≤50% of PAs	>50% of Pas
FHF = focal hepatic fibrosis (LS = liver sample)	≤25% of LS	≤50% of LS	>50% of LS
Hem = hemosiderosis	≤25% of LS	≤50% of LS	>50% of LS
BHF = bile duct hyperplasia/fibrosis	≤25% of PAs	≤50% of PAs	>50% of Pas
LG = lipid granuloma	sum < 7	9 ≤ sum < 15	sum ≥ 15
HIF = hepatic intracellular fat	sum < 7	7 ≤ sum < 15	sum ≥ 15
ITO = Ito/Stellate cell abundance	sum < 20 *	20 ≤ sum < 30 *	sum ≥ 30 *
**Kidney**			
GBMT = glomerular (incl. capsule) basement membrane thickening	<20% of Gl.	<50% of Gl.	≥50% Gl.
GA = glomerular atrophy, Gl = Glomeruli	<10% of Gl.	<25% of Gl.	≥25% Gl.
GMD = glomerular mesangial deposits	<10% of Gl.	<25% Gl.	≥25% Gl.
GHC = glomerular hyper-cellularity	<10% of Gl.	<25% Gl.	≥25% Gl.
DT = dilated tubules	≤50%	<75%	≥75%
THD = intracellular hyaline droplets (PT = proximal tubules)	≤25% of PT	≤50% of PT	>50% of PT
MCI = mononuclear cell infiltration	sum < 9	9 ≤ sum < 20	sum ≥ 20
THC = tubular hyaline casts (CT = collecting tubules)	<10% of CT	<25% CT	≥25% CT
IF = interstitial fibrosis (KS = kidney sample)	<20% of KS	<50% of KS	≥50% KS
GS = glomerular sclerosis	<20% of Gl.	<50% of Gl.	≥50% Gl.
TN = tubular necrosis (Tub. = tubules)	<20% of tub.	<50% of tub.	≥50% tub.

**Table 2:** Biometric data and heavy metal concentrations in liver tissue from Qaanaaq and Qeqertarsuaq ringed seals. *n* = sample size *, SD = standard deviation, Range = min-max. N/A = not analysed, L = liver, ex: Cd-L = liver cadmium concentration.

	Qaanaaq 2008				Qeqertarsuaq 2008			
	*n*	mean	SD	Range	*n*	Mean	SD	Range
Age (years)	19	1.7	2.80	0–11	18	0.53	0.95	0–3
Length (cm)	19	134.2	13.17	108.7–152.8	18	111.65	8.13	97–128
Weight	0	N/A	N/A	N/A	18	28.48	6.90	21–46
Cd-L (μg/g ww)	19	7.80	8.95	0.013–38.79	18	11.58	6.32	0.11–25.45
Hg-L (μg/g ww)	19	3.73	5.01	0.275–23.29	18	1.78	1.70	0.45–8.00
Se-L (μg/g ww)	19	2.43	1.66	0.89–8.12	18	1.54	0.71	0.49–3.78
Molar Hg-L/Se-L	19	0.49	0.21	0.12–1.13	18	0.46	0.16	0.20–0.83

Note: * not all of the 2 × 20 seals were analyzed, as it was not possible to obtain formaldehyde-fixed tissue from all individuals. CD-L: Cadmium liver concentration; Hg-L: Mercury liver concentration; Se-L: Selenium liver concentration.

**Table 3:** Results from the GLM analyses on liver histology. MCI = mononuclear cell infiltration; PCI = portal cell infiltration; Hem. = hemosiderin; FHF = focal hepatic fibrosis; PF = portal fibrosis; BHF = bile duct hyperplasia/fibrosis. −: negative effect.

	Hepatic Histologic Change						
	MCI	PCI	Ito cell abund.	Hem.	FHF	PF	BHF
Age	0.22	0.02 *	0.75	0.803	0.04 *	1.187 × 10^-04^ ***	2.303 × 10^-06^ ***
Hg	0.42	0.20	0.80	2.4 × 10^-05^ *** (−)	0.19	0.85	0.96
Cd	0.56	0.56	0.51	8.7 × 10^-07^ *** (−)	0.17	0.03 *	0.08
Se	0.66	0.60	0.64	4.4 × 10^-04^ *** (−)	0.06	0.42	0.28

*: *p* < 0.05; ***: *p* < 0.001.

**Table 4:** Results from the GLM model on renal histopathology. GBMT = glomerular basement membrane thickening; GA = glomerular atrophy; GMD = glomerular mesangial deposits; GHC = glomerular hyper-cellularity; DT = dilated tubules; THD = tubular hyaline droplets; MCI = mononuclear cell infiltration; THC = tubular hyaline casts; GS = glomerular sclerosis. +: positive effect.

	Renal Histologic Change								
	GBMT	GA	GMD	GHC	DT	THD	MCI	THC	GS
Age	0.07	0.02	0.52	0.04 * (+)	2.0 × 10^-03^ ** (+)	0.08	0.73	0.04 *	0.04 *
Hg	0.99	0.11	0.99	0.09	0.63	0.09	0.17	0.76	0.29
Cd	0.08	0.91	0.82	0.09	0.12	0.33	0.56	0.17	0.87
Se	0.977	0.108	0.859	0.42	0.58	0.13	0.27	0.92	0.15

*: *p* < 0.05; **: *p* < 0.01.

## Data Availability

All data are contained within the article.
